# Antimicrobial and Hypoglycemic Activities of Novel *N*-Mannich Bases Derived from 5-(1-Adamantyl)-4-substituted-1,2,4-triazoline-3-thiones

**DOI:** 10.3390/ijms151222995

**Published:** 2014-12-11

**Authors:** Ebtehal S. Al-Abdullah, Hanaa M. Al-Tuwaijri, Hanan M. Hassan, Mogedda E. Haiba, Elsayed E. Habib, Ali A. El-Emam

**Affiliations:** 1 Department of Pharmaceutical Chemistry, College of Pharmacy, King Saud University, Riyadh 11451, Saudi Arabia; E-Mails: ealabdullah@ksu.edu.sa (E.S.A.-A.); haltuwajri@ksu.edu.sa (H.M.A.-T.); mogedda.haiba@yahoo.com (M.E.H.); 2 Department of Pharmaceutical Sciences, College of Pharmacy, Princess Nourah bint Abdulrahman University, Riyadh 11671, Saudi Arabia; E-Mail: hananhafila@hotmail.com; 3 Department of Therapeutic Chemistry, Pharmaceutical and Drug Industries Division, National Research Center, Tahrir Street, Dokki, Cairo 12622, Egypt; 4 Department of Pharmaceutics and Pharmaceutical Technology, College of Pharmacy, Taibah University, Medinah 11344, Saudi Arabia; E-Mail: sayedhabib@hotmail.co.jp

**Keywords:** adamantane derivatives, 1,2,4-triazoles, *N*-Mannich bases, antimicrobial activity, hypoglycemic activity

## Abstract

The reaction of 5-(1-adamantyl)-4-ethyl or allyl-1,2,4-triazoline-3-thione with formaldehyde solution and various 1-substituted piperazines yielded the corresponding *N*-Mannich bases. The newly synthesized *N*-Mannich bases were tested for *in vitro* inhibitory activities against a panel of Gram-positive and Gram-negative bacteria and the yeast-like pathogenic fungus *Candida albicans*. Six compounds showed potent antibacterial activity against one or more of the tested microorganisms, while two compounds exhibited moderate activity against the tested Gram-positive bacteria. None of the newly synthesized compounds were proved to possess marked activity against *Candida albicans*. The oral hypoglycemic activity of six compounds was determined in streptozotocin (STZ)-induced diabetic rats. Four compounds produced significant strong dose-dependent reduction of serum glucose levels, compared to gliclazide at 10 mg/kg dose level (potency ratio > 75%).

## 1. Introduction

**Figure 1:**
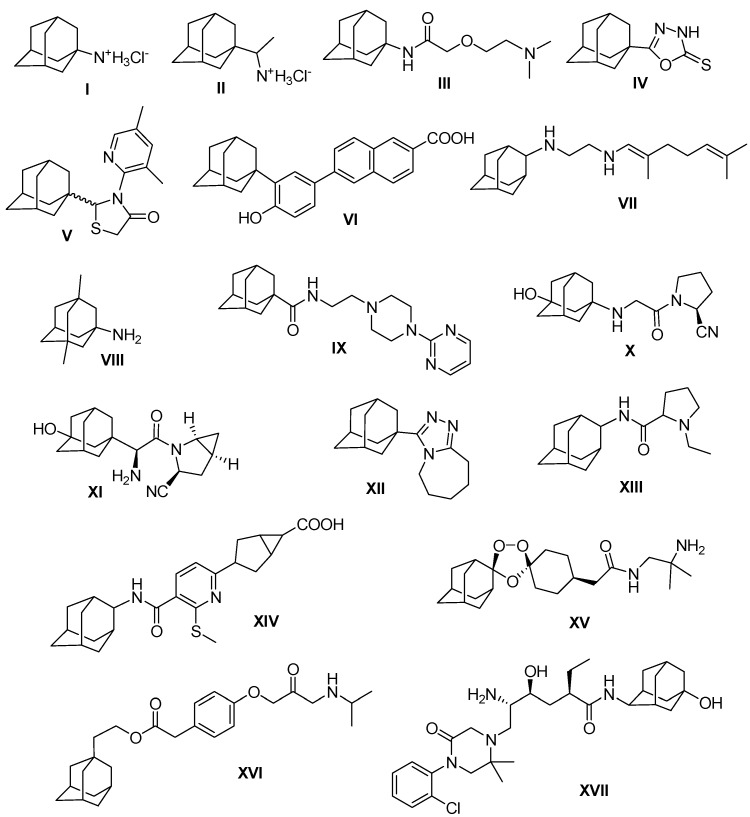
Biologically-active adamantane derivatives.

In almost all cases, an adamantyl-bearing compound will be more lipophilic than the des-adamantyl analogue. Beyond increasing partition coefficient, the adamantyl group positively modulates the therapeutic index of many experimental compounds through a variety of mechanisms [1,2]. Several adamantane derivatives have long been known for their diverse biological activities (Figure 1).

Amantadine (I) was discovered in 1960 as potent antiviral drug for the treatment of influenza A infection [3,4,5] and as an antiparkinsonian drug [6]. Further studies based on amantadine resulted in the discovery of more potent antiviral drugs such as rimantadine (II) [7], and tromantadine (III) [8]. Several adamantane derivatives were also shown to possess marked inhibitory activity against human immunodeficiency viruses (HIV) [9,10,11,12]. The adamantyl-1,3,4-oxadiazoline-2-thione (IV) [11], and the adamantyl-thiazolidin-4-one (V) [12] displayed potent anti-HIV activity. The synthetic retinoid 6-[3-(1-adamantyl)-4-hydroxyphenyl]-2-naphthalene carboxylic acid CD437 (VI) was discovered as a potent inducer of apoptosis in human head and neck squamous cell carcinoma [13]. Several adamantane derivatives were recognized as potent bactericidal and fungicidal agents [14,15,16,17,18,19,20,21,22,23]. SQ109 (VII) was approved for use against drug-susceptible and drug-resistant tuberculosis strains [23]. SQ109 also showed excellent inhibitory activity against *helicobacter pylori* related duodenal ulcers and carcinomas, and *Candida glabrata* [24]. Memantine (VIII), the dimethyl analogue of amantadine, is used as effective therapy for treatment of moderate to severe Alzheimer’s disease [25]. Adatanserin (IX) is a mixed 5-HT_A_ receptor agonist and also has neuroprotective effects against ischemia-induced glutamatergic excitotoxicity [26]. Vildagliptin (X) [27] and Saxagliptin (XI) [28] are members of a new class of oral hypoglycemic agents known as dipeptidyl peptidase IV (DPP-IV) inhibitors, which were approved for the treatment of type 2 diabetes [29]. Adamantane derivatives constitutes the major class of 11β-hydroxysteroid dehydrogenase type 1 (11β-HSD1) inhibitor, which are considered important therapy for controlling non-insulin-dependent diabetes, hyperglycemia, obesity, insulin resistance, hyperlipidemia, hypertension and other symptoms associated with excessive body cortisol [30,31]. MK-544 (XII) [32], PF-877423 (XIII) [33], AZD6925 (XIV) [34], are recently developed drug candidates for the treatment of non-insulin-dependent diabetes and obesity. Moreover, anti-inflammatory activity was reported in several adamantane-containing molecules [19,20,21,22,35,36,37,38]. Arterolane (XV) [39], adaprolol (XVI) [40] and DS-8108b (XVII) [41] are recently developed adamantane-containing drugs for the treatment of malaria, glaucoma and hypertension, respectively.

In continuation of our interest in the chemical and pharmacological properties of adamantane derivatives [11,18,19,20,21,22,35], we report herein the synthesis of new series of 5-(1-adamantyl)-4-substituted-1,2,4-triazole *N*-Mannich bases, and their *in vitro* antimicrobial and *in vivo* hypoglycemic activities.

## 2. Results and Discussion

### 2.1. Chemistry

**Scheme 1:**
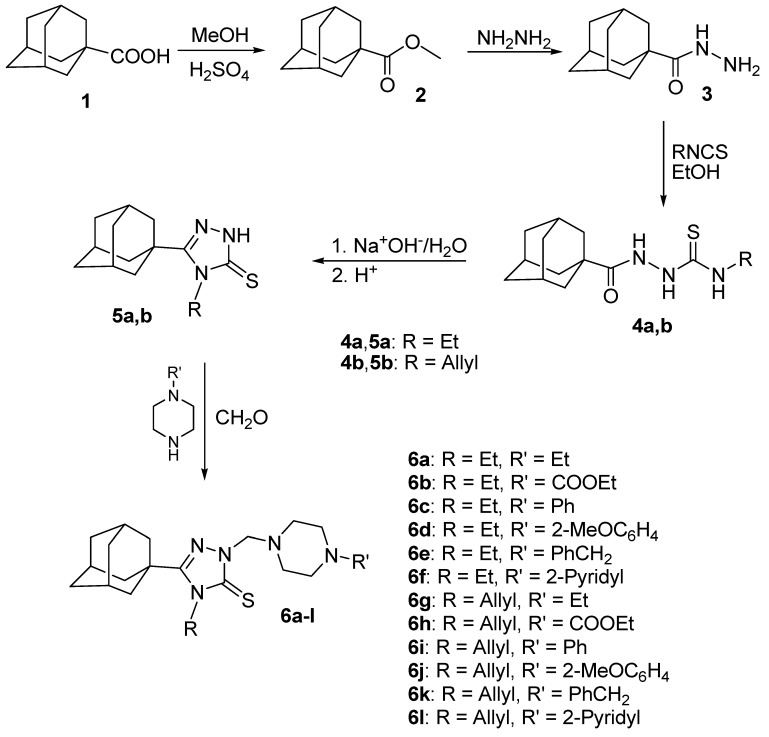
Synthesis the target *N*-Mannich bases **6a**–**l**.

Adamantane-1-carbohydrazide **3**, required as starting material, was obtained via esterification of adamantane-1-carboxylic acid **1** with methanol to get the methyl ester **2**, which was subsequently heated with hydrazine to afford the target carbohydrazide **3** [35]. The reaction of compound **3** with ethyl or allyl isothiocyanate yielded the intermediate 1-(1-adamantylcarbonyl)-4-substituted thiosemicarbazides **4a** and **4b**, which were cyclized to the corresponding 5-(1-adamantyl)-4-substituted-1,2,4-triazoline-3-thiones **5a** [35] and **5b** [19] via heating in 10% aqueous sodium hydroxide. Compounds **5a**,**b** were reacted with the corresponding 1-substituted piperazine and formaldehyde solution in ethanol to yield the corresponding *N*-Mannich bases **6a**–**l** in good to moderate yields. The reaction was carried out by heating the reactants in ethanol for 15 min to enhance the solubility of compounds **5a**,**b** (Scheme 1, Table 1). The structures of compounds **6a**–**l** were confirmed by elemental analyses, in addition to the ^1^H NMR, ^13^C NMR, and electrospray ionization (ESI-MS) or electron impact (EI-MS) mass spectral data which were in full agreement with their structures, and the X-ray spectra of compounds **6c** [42], **6d** [43] and **6e** [44].

**Table 1:** Crystallization solvents (Cryst. Solv.), melting points (m.p.), yield percentages, molecular formulae and molecular weights (Mol. Wt.) of compounds **6a**–**l**.

Comp. No.	*R*	*R*'	Cryst. Solv.	m.p. (°C)	Yield (%)	Molecular Formula (Mol. Wt.)
**6a**	C_2_H_5_	C_2_H_5_	EtOH/H_2_O	158–160	77	C_21_H_35_N_5_S (389.6)
**6b**	C_2_H_5_	COOC_2_H_5_	EtOH/H_2_O	153–155	72	C_22_H_35_N_5_O_2_S (433.61)
**6c**	C_2_H_5_	C_6_H_5_	EtOH	139–141	88	C_25_H_35_N_5_S (437.64)
**6d**	C_2_H_5_	2-CH_3_OC_6_H_4_	EtOH	204–206	92	C_26_H_37_N_5_OS (467.67)
**6e**	C_2_H_5_	C_6_H_5_CH_2_	EtOH	168–170	81	C_26_H_37_N_5_S (451.67)
**6f**	C_2_H_5_	2-Pyridyl	EtOH/H_2_O	144–146	92	C_24_H_34_N_6_S (438.63)
**6g**	CH_2_=CHCH_2_	C_2_H_5_	EtOH/H_2_O	109–111	66	C_22_H_35_N_5_S (401.61)
**6h**	CH_2_=CHCH_2_	COOC_2_H_5_	EtOH/H_2_O	114–116	60	C_23_H_35_N_5_O_2_S (445.62)
**6i**	CH_2_=CHCH_2_	C_6_H_5_	EtOH	161–163	69	C_26_H_35_N_5_S (449.65)
**6j**	CH_2_=CHCH_2_	2-CH_3_OC_6_H_4_	EtOH	208–210	74	C_27_H_37_N_5_OS (479.68)
**6k**	CH_2_=CHCH_2_	C_6_H_5_CH_2_	EtOH/H_2_O	109–111	59	C_27_H_37_N_5_S (463.68)
**6l**	CH_2_=CHCH_2_	2-Pyridyl	EtOH/H_2_O	133–135	66	C_25_H_34_N_6_S (450.64)

### 2.2. In Vitro Antimicrobial Activity

The newly synthesized compounds **6a**–**l** were tested for their *in vitro* growth inhibitory activity against the standard strains of the Institute of Fermentation of Osaka (IFO) namely; *Staphylococcus aureus* IFO 3060, *Bacillus subtilis* IFO 3007, *Micrococcus luteus* IFO 3232 (Gram-positive bacteria), *Escherichia coli* IFO 3301, *Pseudomonas aeuroginosa* IFO 3448 (Gram-negative bacteria), and the yeast-like pathogenic fungus *Candida albicans* IFO 0583. The primary screening was carried out using the agar disc-diffusion method using Müller-Hinton agar medium [45]. The results of the preliminary antimicrobial testing of compounds **6a**–**l** (200 μg/disc), the antibacterial antibiotics Ampicillin trihydrate, Gentamicin (100 μg/disc) and the antifungal drug Clotrimazole (100 μg/disc) and the calculated log *p* values (Clog *p*) of the tested compounds (calculated using the CS ChemOffice Ultra version 8.0, CambridgeSoft, Cambridge, MA, USA) are shown in Table 2.

**Table 2:** Antimicrobial activity of compounds **6a**–**l** (200 μg/8 mm disc), the broad spectrum antibacterial drugs Gentamicin (100 μg/8 mm disc), Ampicillin (100 μg/8 mm disc) and the antifungal drug Clotrimazole (100 μg/8 mm disc) against *Staphylococcus aureus* IFO 3060 (*SA*), *Bacillus subtilis* IFO 3007 (*BS*), *Micrococcus luteus* IFO 3232 (*ML*), *Escherichia coli* IFO 3301 (*EC*), *Pseudomonas aeuroginosa* IFO 3448 (*PA*), and *Candida albicans* IFO 0583 (*CA*).

Comp. No.	Clog *p*	Diameter of Growth Inhibition Zone (mm) ^a^					
		*SA*	*BS*	*ML*	*EC*	*PA*	*CA*
**6a**	3.96	–	–	–	–	–	–
**6b**	5.51	–	–	–	–	–	–
**6c**	5.67	–	–	–	–	–	–
**6d**	5.69	15	17	11	–	–	–
**6e**	6.59	21 (8) ^b^	20 (4) ^b^	17	14	–	–
**6f**	4.72	15	18 (8) ^b^	11	–	–	–
**6g**	4.21	11	13	–	–	–	–
**6h**	5.76	–	12	–	–	–	–
**6i**	5.92	14	16	12	–	–	–
**6j**	5.82	19 (8) ^b^	18 (16) ^b^	18 (16) ^b^	–	–	–
**6k**	6.84	28 (0.5) ^b^	30 (0.5) ^b^	19 (8) ^b^	18 (16) ^b^	12	–
**6l**	4.97	17	19 (8) ^b^	10	–	–	–
**Gentamicin**		26 (2) ^b^	25 (2) ^b^	18 (2) ^b^	20 (0.5) ^b^	19 (1) ^b^	NT
**Ampicillin**		23 (2) ^b^	21 (0.5) ^b^	19 (2) ^b^	17 (2) ^b^	16 (2) ^b^	NT
**Clotrimazole**		NT	NT	NT	NT	NT	21 (2) ^b^

^a^ (–): Inactive (inhibition zone < 10 mm); NT: Not tested. ^b^ The figures shown in parentheses represent the minimal inhibitory concentration (MIC) values (μg/mL).

The results revealed that the tested compounds showed varying degrees of inhibition against the tested microorganisms. Strong antibacterial activity was displayed by the compounds **6d**, **6e**, **6f**, **6j**, **6k** and **6l** which produced growth inhibition zones ≥18 mm against one or more of the tested microorganisms. Meanwhile, the compounds **6c** and **6i** showed moderate activity (growth inhibition zones 14–17 mm), the compounds **6g** and **6h** produced marginal activity (growth inhibition zones 10–13 mm) and compounds **6a** and **6b** were practically inactive (growth inhibition zones < 10 mm) against the tested microorganisms.

The Gram-positive bacteria *Bacillus subtilis* and *Staphylococcus aureus* and to a lesser extent *Micrococcus luteus* are considered the most sensitive among the tested microorganisms. The activity against the tested Gram-negative bacteria was generally lower than that against the Gram-positive bacteria; compound **6d** and **6k** were strongly active against *Escherichia coli* and weakly active against *Pseudomonas aeuroginosa*. The newly synthesized compounds **6a**–**l** lacked inhibitory activity against *Candida albicans*.

It was observed that the antibacterial activity of the newly synthesized *N*-Mannich bases **6a**–**l** was mainly dependent on the nature of 4-piperazine substituents regardless the nature of the 1,2,4-triazole substituent at position 4 whether an ethyl or allyl. The presence of 4-aryl, benzyl or 2-pyridyl at the piperazine *N*-4 was optimistic for antibacterial activity, whereas, the aliphatic substituents (C_2_H_5_, COOC_2_H_5_) deteriorated the antibacterial activity. The lipophilicity of the compounds **6a**–**l** had limited effect on the antibacterial activity. The minimal inhibitory concentrations (MIC) [46] for the most active compounds **6d**, **6e**, **6f**, **6j**, **6k** and **6l**, which are shown in Table 2, were in accordance with the results obtained in the primary antimicrobial screening.

### 2.3. In Vivo Hypoglycemic Activity

The oral hypoglycemic activity of compounds **6a**, **6c**, **6f**, **6g**, **6j** and **6l** was determined in streptozotocin (STZ)-induced diabetic rats. The compounds were tested at 10 and 20 mg/kg dose levels. The diabetogenic effect of STZ is the direct result of irreversible damage to the pancreatic beta cells, resulting in degranulation and loss of insulin secretion [47,48].

The results of oral hypoglycemic activity of compounds **6a**, **6c**, **6f**, **6g**, **6j** and **6l** (10 and 20 mg/kg) and the potent hypglycemic drug gliclazide in STZ-induced diabetic rats (10 mg/kg) are listed in Table 3. The highest activity was shown by compounds **6a**, **6f**, **6g** and **6l**, which produced significant strong dose-dependent reduction of serum glucose levels in STZ-induced diabetic rats, compared to gliclazide at 10 mg/kg dose level (potency ratio > 75%). Compound **6j** displayed good hypoglycemic activity at 20 mg/kg dose level and a weak activity at 10 mg/kg dose level. Compound **6c** produced medium activity at 10 mg/kg dose level (potency ratio 40.66%), without significant increase at 20 mg/kg dose level. The structure-hypoglycemic activity relationship of the tested adamantyl 1,2,4-triazoles *N*-Mannich bases revealed that the hypoglycemic activity is mainly dependent on the nature of the piperazine *N*-4 substituents. The piperazine *N*-4 ethyl and 2-pyridyl substituents were optimistic (compounds **6a**, **6f**, **6g** and **6l**), while the aromatic substituents (phenyl and 2-methoxyphenyl) were less active at 10 mg/kg dose level and retained the good activity at 20 mg/kg doses (compounds **6c** and **6j**). In addition, the 4-triazole substituents (ethyl and allyl) showed no significant influence on the hypoglycemic activity.

**Table 3:** Oral hypoglycemic activity of compounds **6a**, **6c**, **6f**, **6g**, **6j** and **6l** (10 and 20 mg/kg) and gliclazide (10 mg/kg) in streptozotocin (STZ)-induced diabetic rats.

Treatment	Results		
	C_0_ (mg/dL) ^a^	C_24_ (mg/dL) ^a^	% Glucose Reduction ^b^
**Group 1** ^c^	302.6 ± 11.64	287.2 ± 16.85	5.09%
**Group 2** ^d^	295.4 ± 17.52	183.0 ± 13.38 *	38.05%
**6a** (10 mg/kg)	289.0 ± 18.05	204.4 ± 7.37 *	29.27% (76.93%)
**6a** (20 mg/kg)	290.4 ± 10.60	188.0 ± 8.6 *	35.26% (46.34%)
**6c** (10 mg/kg)	285.8 ± 14.31	241.6 ± 19.2	15.47% (40.66%)
**6c** (20 mg/kg)	297.6 ± 18.57	245.4 ± 11.54	17.54% (23.05%)
**6f** (10 mg/kg)	289.0 ± 14.35	197.8 ± 6.13 *	31.56% (82.94%)
**6f** (20 mg/kg)	292.6 ± 10.33	189.0 ± 7.42 *	35.41% (46.53%)
**6g** (10 mg/kg)	291.0 ± 5.58	190.6 ± 9.05 *	34.50% (90.67%)
**6g** (20 mg/kg)	283.4 ± 10.45	184.0 ± 15.28 *	35.07% (46.09%)
**6j** (10 mg/kg)	278.4 ± 15.07	228.4 ± 11.41	17.96% (47.20%)
**6j** (20 mg/kg)	297.4 ± 17.94	203.4 ± 15.28 *	31.61% (41.54%)
**6l** (10 mg/kg)	290.4 ± 18.37	199.0 ± 18.75 *	31.47% (82.71%)
**6l** (20 mg/kg)	295.4 ± 12.58	200.0 ± 18.78 *	32.30% (42.45%)

^a^ Results are expressed as mean ± SEM. (*n* = 5); ^b^ The figures shown in parentheses are the relative potency compared with glicalzide; ^c^ Treated with a single oral dose of 0.5% (*w*/*v*) aqueous CMC solution (5 mL/kg); ^d^ Treated with 10 mg/kg gliclazide in 0.5% (*w*/*v*) aqueous CMC; and * Significant difference at *p* < 0.01 compared with the corresponding control.

### 2.4. Oral Acute Toxicity Testing

The method of Litchfield and Wilcoxon was adopted for measuring the acute oral toxicity of compounds **6a**, **6f**, **6g** and **6l** which possessed the highest hypoglycemic activity [49]. The acute toxicity results of compounds **6a**, **6f**, **6g** and **6l** in normal albino mice are listed in Table 4. The oral LD_50_ of gliclazide was reported to be >3000 mg/kg in mice [50]. Although the oral acute toxicity of the tested adamantyl 1,2,4-triazole derivatives is higher than that of gliclazide, the compounds induce their hypoglycemic activity at safe doses.

**Table 4:** Oral acute toxicity (mg/kg) of compounds **6a**, **6f**, **6g** and **6l** in normal albino mice.

Comp. No.	LD_50_ *	LD_50_ (95% Confidence Limit)
**6a**	892 ± 38.70	892 (844–1089)
**6f**	787 ± 41.05	787 (721–923)
**6g**	833 ± 23.80	833 (765–891)
**6l**	741 ± 22.50	741 (624–806)

* Results are expressed as mean ± SEM. (*n* = 6).

## 3. Experimental Section

### 3.1. General

Melting points (°C) were measured in open glass capillaries using a Branstead 9100 Electrothermal melting point apparatus (Thermo Fisher Scientific, Waltham, MA, USA) and are uncorrected. NMR spectra were obtained on a Bruker AC 500 Ultra Shield NMR spectrometer (Bruker, Fällanden, Switzerland) operating at 500.13 MHz for ^1^H and 125.76 MHz for ^13^C, the chemical shifts are expressed in δ (ppm) downfield from tetramethylsilane (TMS) as internal standard; Coupling constants (*J*) are expressed in Hz. Electrospray ionization mass spectra (ESI-MS) were recorded on an Agilent 6410 Triple Quad tandem mass spectrometer (Agilent Technologies, Santa Clara, CA, USA) at 4.0 and 3.5 kV for positive and negative ions, respectively. High resolution mass spectra (HR-MS) were recorded on JEOL JMS-700 (JEOL, Tokyo, Japan) using Electron Impact (EI) ionization mode by keeping ionization energy at 70 eV. Elemental analyses (C, H, N and S) were in agreement with the proposed structures within ±0.4% of the theoretical values. Monitoring the reactions and checking the purity of the final products were carried out by thin layer chromatography (TLC) using silica gel precoated aluminum sheets (60 F_254_, Merck, Darmstadt, Germany) and visualization with ultraviolet light (UV) at 365 and 254 nm. The bacterial strains and *Candida albicans* fungus were obtained from the Institute of Fermentation of Osaka (IFO), Osaka, Japan. The reference drugs Ampicillin trihydrate (CAS 7177-48-2), Gentamicin sulfate (CAS 1405-41-0), Clotrimazole (CAS 23593-75-1) and Gliclazide (CAS 21187-98-4) were purchased from Sigma-Aldrich Chemie GmbH, Taufkirchen, Germany. The Sprague-Dawley rats and the normal albino mice were purchased from a local animal house (Abu-Rawash, Giza, Egypt). The animal experiments for the determination of the hypoglycemic activity and acute toxicity were performed according to the Ethics Committee of the National Research Centre and in accordance with the recommendations for the proper care and use of laboratory animals “Canadian Council on Animal Care Guidelines, 1984”.

### 3.2. General Procedure for the Preparation of 5-(1-Adamantyl)-4-ethyl or allyl-2-(4-substituted piperazine-1-ylmethyl)-1,2,4-triazoline-3-thiones **6a**–**l**

A mixture of compound **5a** or **5b** (2.0 mmol), the appropriate *N*-substituted piperazine (2.0 mmol) and 37% formaldehyde solution (0.5 mL), in ethanol (8 mL), was heated under reflux for 15 min when a clear solution was obtained. Stirring was continued for 12 h at room temperature and the mixture was allowed to stand overnight. Cold water (5 mL) was added and the reaction mixture was stirred for 20 min. The precipitated crude products were filtered, washed with water, dried, and crystallized from ethanol or aqueous ethanol.

**6a**: ^1^H NMR (CDCl_3_): δ 1.29–1.36 (m, 6H, CH_2_C***H***_3_), 1.69–1.72 (m, 6H, Adamantane-H), 1.98 (s, 6H, Adamantane-H), 2.01 (s, 3H, Adamantane-H), 2.60–2.94 (m, 8H, Piperazine-H), 4.14–4.21 (m, 4H, C***H***_2_CH_3_), 5.05 (s, 2H, CH_2_). ^13^C NMR: δ 13.75, 16.77 (CH_2_***C***H_3_), 27.97, 35.27, 36.30, 39.82 (Adamantane-C), 41.07, 41.63 (***C***H_2_CH_3_), 48.87, 52.26 (Piperazine-C), 68.34 (CH_2_), 157.97 (Triazole C-5), 168.73 (C=S). ESI-MS, *m*/*z*: 390.3 (M + H)^+^. EI-HRMS, *m*/*z* (Rel. Int.): 391.9701 (15.35), 390.9654 (72.55), 389.9709 (100), 263.1793 (6.89), 233.9379 (16.86), 229.9876 (49.0), 221.9822 (30.64), 220.9799 (16.40), 219.9868 (71.11), 208.0032 (22.56), 205.9923 (66.25), 165.9672 (13.29), 155.9966 (21.69), 135.9874 (20.40), 127.0312 (59.65), 125.0127 (19.34), 112.9976 (12.81), 83.9997 (71.21), 76.9676 (26.56).

**6b**: ^1^H NMR (CDCl_3_): δ 1.17 (t, 3H, CH_2_C***H***_3_, *J* = 7.0 Hz), 1.33 (t, 3H, CH_2_C***H***_3_, *J* = 7.0 Hz), 1.69–1.76 (m, 6H, Adamantane-H), 1.98 (s, 6H, Adamantane-H), 2.06 (s, 3H, Adamantane-H), 2.73 (br. s, 4H, Piperazine-H), 3.45 (br. s, 4H, Piperazine-H), 4.02 (q, 2H, C***H***_2_CH_3_, *J* = 7.0 Hz), 4.17 (q, 2H, C***H***_2_CH_3_, *J* = 7.0 Hz), 5.07 (s, 2H, CH_2_). ^13^C NMR: δ 12.73 (CH_2_***C***H_3_), 13.62 (CH_2_***C***H_3_), 26.92, 34.28, 35.26, 38.82 (Adamantane-C), 40.43 (***C***H_2_CH_3_), 44.93, 49.25 (Piperazine-C), 60.40 (***C***H_2_CH_3_), 71.50 (CH_2_), 154.38 (C=O), 155.38 (Triazole C-5), 166.33 (C=S). ESI-MS, *m*/*z*: 434.3 (M + H)^+^. EI-HRMS, *m*/*z* (Rel. Int.): 433.9816 (12.48), 432.9806 (41.65), 388.7850 (9.89), 276.0345 (7.42), 263.9471 (33.73), 262.9412 (71.36), 189.0572 (22.73), 172.0117 (92.34), 171.0052 (100), 169.9955 (51.80), 135.0313 (47.86), 127.0339 (65.01), 96.9849 (27.69), 83.9934 (10.11), 70.0012 (20.73).

**6c**: ^1^H NMR (CDCl_3_): δ 1.13 (t, 3H, CH_2_C***H***_3_, *J* = 7.0 Hz), 1.67-1.73 (m, 6H, Adamantane-H), 1.96 (s, 6H, Adamantane-H), 2.03 (s, 3H, Adamantane-H), 2.88 (s, 4H, Piperazine-H), 3.09 (s, 4H, Piperazine-H), 4.17 (q, 2H, C***H***_2_CH_3_, *J* = 7.0 Hz), 5.08 (s, 2H, CH_2_), 6.46–6.83 (m, 3H, Ar-H), 7.15–7.17 (m, 2H, Ar-H). ^13^C NMR: δ 13.81 (CH_2_***C***H_3_), 27.95, 35.24, 36.31, 39.90 (Adamantane-C), 43.43 (***C***H_2_CH_3_), 49.40, 50.37 (Piperazine-C), 68.80 (CH_2_), 116.32, 119.99, 129.12, 151.27 (Ar-C), 156.10 (Triazole C-5), 168.75 (C=S). ESI-MS, *m*/*z*: 438.3 (M + H)^+^.

**6d**: ^1^H NMR (CDCl_3_): δ 1.32 (t, 3H, CH_2_C***H***_3_, *J* = 7.0 Hz), 1.71–1.76 (m, 6H, Adamantane-H), 1.98–2.12 (m, 9H, Adamantane-H), 3.08 (s, 8H, Piperazine-H), 3.81 (s, 3H, OCH_3_), 4.15 (q, 2H, C***H***_2_CH_3_, *J* = 7.0 Hz), 5.15 (s, 2H, CH_2_), 6.79–7.01 (m, 4H, Ar-H). ^13^C NMR: δ 13.76 (CH_2_***C***H_3_), 27.92, 35.32, 36.48, 39.83 (Adamantane-C), 43.83 (***C***H_2_CH_3_), 47.40, 50.18 (Piperazine-C), 55.48 (OCH_3_), 72.58 (CH_2_), 111.43, 118.38, 121.12, 123.55, 152.13, 152.26 (Ar-C), 156.57 (Triazole C-5), 167.34 (C=S). ESI-MS, *m*/*z*: 468.4 (M + H)^+^. EI-HRMS, *m*/*z* (Rel. Int.): 469.0323 (4.53), 468.0119 (11.48), 263.7015 (23.50), 262.6953 (10.46), 261.6985 (11.70), 205.9870 (70.30), 203.9838 (45.34), 202.9813 (34.01), 190.0338 (54.50), 162.0190 (39.20), 136.0422 (12.45), 135.0388 (82.48), 133.9844 (49.53), 119.9954 (30.67), 107.0433 (6.26), 90.9758 (15.70), 78.9755 (22.69), 76.9620 (19.90), 70.0146 (100), 64.9748 (9.53), 55.9825 (14.75).

**6e**: ^1^H NMR (CDCl_3_): δ 1.13 (t, 3H, CH_2_C***H***_3_, *J* = 6.5 Hz), 1.68–1.73 (m, 6H, Adamantane-H), 1.98–2.10 (m, 9H, Adamantane-H), 2.72–2.76 (m, 4H, Piperazine-H), 3.24–3.26 (m, 4H, Piperazine-H), 3.72 (s, 2H, Benzylic-CH_2_), 4.15 (q, 2H, C***H***_2_CH_3_, *J* = 6.5 Hz), 5.02 (s, 2H, CH_2_), 7.20–7.54 (m, 5H, Ar-H). ^13^C NMR: δ 12.74 (CH_2_***C***H_3_), 26.90, 34.36, 35.26, 38.82 (Adamantane-C), 40.62 (***C***H_2_CH_3_), 46.01, 50.37 (Piperazine-C), 61.59 (Benzylic-**C**H_2_), 66.66 (CH_2_), 127.40, 128.31, 129.19, 130.57 (Ar-C), 155.55 (Triazole C-5), 167.73 (C=S). ESI-MS, *m*/*z*: 452.3 (M + H)^+^. EI-HRMS, *m*/*z* (Rel. Int.): 451.6757 (2.35), 163.8833 (8.26), 262.8768 (27.15), 219.9799 (3.46), 205.9263 (4.21), 190.0603 (35.75), 189.0528 (100), 188.0480 (29.73), 146.0319 (7.87), 135.0343 (17.63), 131.9975 (4.73), 96.9961 (8.44), 91.9824 (8.09), 90.9752 (74.21), 76.9642 (4.83), 64.9756 (7.23).

**6f**: ^1^H NMR (CDCl_3_): δ 1.41 (t, 3H, CH_2_C***H***_3_, *J* = 7.0 Hz), 1.77–1.83 (m, 6H, Adamantane-H), 2.06 (s, 6H, Adamantane-H), 2.12 (s, 3H, Adamantane-H), 2.92 (t, 4H, Piperazine-H, *J* = 5.0 Hz), 3.57 (br. s, 4H, Piperazine-H), 4.25 (q, 2H, C***H***_2_CH_3_, *J* = 7.0 Hz), 5.18 (s, 2H, CH_2_), 6.62–6.66 (m, 2H, Pyridine-H), 7.48–7.50 (m, 1H, Pyridine-H), 8.18 (d, 1H, Pyridine-H, *J* = 3.5 Hz). ^13^C NMR: δ 13.79 (CH_2_***C***H_3_), 27.94, 36.29, 37.09, 39.29 (Adamantane-C), 41.58 (***C***H_2_CH_3_), 45.35, 50.24 (Piperazine-C), 68.92 (CH_2_), 107.35, 113.25, 137.83, 148.03, 156.11 (Pyridine-C), 158.99 (Triazole C-5), 167.73 (C=S). ESI-MS, *m*/*z*: 439.3 (M + H)^+^. EI-HRMS, *m*/*z* (Rel. Int.): 439.8562 (3.34), 438.8099 (10.97), 263.9348 (9.29), 262.9257 (33.99), 229.9813 (5.54), 177.0416 (47.75), 176.0352 (100), 175.02686 (32.67), 147.0295 (37.10), 135.0451 (12.27), 133.0068 (12.67), 121.0062 (48.64), 107.0549 (14.98), 95.0212 (13.15), 78.9646 (9.30), 77.9577 (14.59).

**6g**: ^1^H NMR (CDCl_3_): δ 1.11 (t, 3H, CH_2_C***H***_3_, *J* = 7.2 Hz), 1.77–1.79 (m, 6H, Adamantane-H), 2.01–2.10 (m, 9H, Adamantane-H), 2.51 (br. s, 8H, Piperazine-H), 2.92 (q, 2H, C***H***_2_CH_3_, *J* = 7.2 Hz), 4.88–4.93 (m, 2H, –C***H***_2_–CH=), 5.01–5.02 (m, 1H, C***H***_2_=CH), 5.14–5.20 (m, 3H, C***H***_2_=CH & CH_2_), 5.90–5.92 (m, 1H, CH_2_=C***H***). ^13^C NMR: δ 13.55 (CH_2_***C***H_3_), 28.05, 35.87, 36.37, 40.0 (Adamantane-C), 49.35 (–***C***H_2_–CH=), 49.85, 51.65 (Piperazine-C), 71.75 (CH_2_), 116.95 (***C***H_2_=CH), 132.65 (CH_2_=***C***H), 155.85 (Triazole C-5), 168.25 (C=S). ESI-MS, *m*/*z*: 402.4 (M + H)^+^.

**6h**: ^1^H NMR (CDCl_3_): δ 1.15 (t, 3H, CH_2_C***H***_3_, *J* = 7.0 Hz), 1.67–1.74 (m, 6H, Adamantane-H), 1.96 (s, 6H, Adamantane-H), 2.03 (s, 3H, Adamantane-H), 2.67–2.69 (m, 4H, Piperazine-H), 3.39–3.41 (m, 4H, Piperazine-H), 4.02 (q, 2H, C***H***_2_CH_3_, *J* = 7.0 Hz), 4.83–4.84 (m, 2H, –C***H***_2_–CH=), 4.87–4.90 (m, 1H, C***H***_2_=CH), 5.06 (s, 2H, CH_2_), 5.20–5.22 (m, 1H, C***H***_2_=CH), 5.80–5.86 (m, 1H, CH_2_=C***H***) ^13^C NMR: δ 14.62 (CH_2_***C***H_3_), 27.90, 35.34, 36.26, 39.91 (Adamantane-C), 43.63, 50.27 (Piperazine-C), 47.63 (–***C***H_2_–CH=), 61.53 (***C***H_2_CH_3_), 69.23 (CH_2_), 117.56 (***C***H_2_=CH), 131.62 (CH_2_=***C***H), 155.50 (C=O), 156.56 (Triazole C-5), 169.48 (C=S). ESI-MS, *m*/*z*: 446.3 (M + H)^+^.

**6i**: ^1^H NMR (CDCl_3_): δ 1.66–1.74 (m, 6H, Adamantane-H), 1.96–2.09 (m, 9H, Adamantane-H), 3.20 (br. s, 4H, Piperazine-H), 3.49 (br. s, 4H, Piperazine-H), 4.81–4.85 (m, 2H, –C***H***_2_–CH=), 4.95–5.01 (m, 1H, C***H***_2_=CH), 5.22–5.26 (m, 3H, C***H***_2_=CH & CH_2_), 5.80–5.83 (m, 1H, CH_2_=C***H***), 6.90–6.98 (m, 2H, Ar-H), 7.23–7.38 (m, 3H, Ar-H). ^13^C NMR: δ 27.89, 35.48, 36.26, 39.87 (Adamantane-C), 43.30, 48.07 (Piperazine-C), 48.52 (–***C***H_2_–CH=), 72.73 (CH_2_), 117.47 (***C***H_2_=CH), 129.46 (CH_2_=***C***H), 115.42, 118.12, 139.46, 149.06 (Ar-C), 156.92 (Triazole C-5), 168.05 (C=S). ESI-MS, *m*/*z*: 450.3 (M + H)^+^.

**6j**: ^1^H NMR (CDCl_3_): δ 1.69–1.74 (m, 6H, Adamantane-H), 1.96–1.98 (m, 6H, Adamantane-H), 2.04 (s, 3H, Adamantane-H), 2.90–3.20 (m, 8H, Piperazine-H), 3.81 (s, 3H, OCH_3_), 4.79–4.85 (m, 2H, –C***H***_2_–CH=), 4.95–5.01 (m, 1H, C***H***_2_=CH), 5.17 (s, 2H, CH_2_), 5.22–5.24 (m, 1H, C***H***_2_=CH), 5.80–5.87 (m, 1H, CH_2_=C***H***), 6.79–6.99 (m, 4H, Ar-H). ^13^C NMR: δ 27.94, 35.48, 36.30, 39.86 (Adamantane-C), 43.80, 50.61 (Piperazine-C), 47.56 (–***C***H_2_–CH=), 55.48 (OCH_3_), 72.72 (CH_2_), 118.12 (***C***H_2_=CH), 131.05 (CH_2_=***C***H), 110.90, 111.43, 118.98, 121.13, 152.15, 152.26 (Ar-C), 156.91 (Triazole C-5), 168.06 (C=S). ESI-MS, *m*/*z*: 480.4 (M + H)^+^. EI-HRMS, *m*/*z* (Rel. Int.): 479.6629 (1.54), 275.6026 (1.07), 240.3820 (2.66), 235.9632 (26.93), 206.0158 (7.68), 203.9816 (6.69), 189.9879 (6.09), 162.0124 (100), 171.0076 (17.38), 160.0030 (8.43), 149.0092 (2.29), 135.0312 (4.97), 133.9789 (3.35), 119.9954 (2.95), 69.9917 (10.89).

**6k**: ^1^H NMR (CDCl_3_): δ 1.65–1.73 (m, 6H, Adamantane-H), 1.94 (s, 6H, Adamantane-H), 2.02 (s, 3H, Adamantane-H), 2.42 (br. s, 4H, Piperazine-H), 2.78 (br. s, 4H, Piperazine-H), 3.45 (s, 2H, Benzylic C***H***_2_), 4.82 (s, 2H, –C***H***_2_–CH=), 4.91–4.94 (m, 1H, C***H***_2_=CH), 5.19–5.21 (m, 1H, C***H***_2_=CH), 5.05 (s, 2H, CH_2_), 5.80–5.83 (m, 1H, CH_2_=C***H***), 7.18–7.23 (m, 5H, Ar-H). ^13^C NMR: δ 27.92, 33.29, 36.30, 39.90 (Adamantane-C), 48.22, 52.99 (Piperazine-C), 50.20 (–***C***H_2_–CH=), 58.31 (Benzylic ***C***H_2_), 69.12 (CH_2_), 117.71 (***C***H_2_=CH), 127.22, 128.25, 129.43, 137.52 (Ar-C), 131.46 (CH_2_=***C***H), 156.36 (Triazole C-5), 169.42 (C=S). ESI-MS, *m*/*z*: 464.4 (M + H)^+^.

**6l**: ^1^H NMR (CDCl_3_): δ 1.65–1.73 (m, 6H, Adamantane-H), 1.93 (s, 6H, Adamantane-H), 2.02 (s, 3H, Adamantane-H), 2.84–2.86 (m, 4H, Piperazine-H), 3.46–3.48 (m, 4H, Piperazine-H), 4.83–4.84 (m, 2H, –C***H***_2_–CH=), 4.85–4.89 (m, 1H, C***H***_2_=CH), 5.14 (s, 2H, CH_2_), 5.18–5.20 (m, 1H, C***H***_2_=CH), 5.81–5.83 (m, 1H, CH_2_=C***H***), 6.53–6.67 (m, 2H, Pyridine-H), 7.38–7.42 (m, 1H, Pyridine-H), 8.10 (d, 1H, Pyridine-H, *J* = 2.5 Hz). ^13^C NMR: δ 27.91, 35.32, 36.28, 39.93 (Adamantane-C), 45.33, 50.26 (Piperazine-C), 48.16 (–***C***H_2_–CH=), 69.16 (CH_2_), 117.54 (***C***H_2_=CH), 131.41 (CH_2_=***C***H), 107.26, 113.32, 137.68, 147.66, 159.29 (Pyridine-C), 156.48 (Triazole C-5), 169.48 (C=S). ESI-MS, *m*/*z*: 451.4 (M + H)^+^. EI-HRMS, *m*/*z* (Rel. Int.): 451.8970 (5.24), 450.9609 (15.48), 277.9037 (4.19), 276.9116 (15.45), 275.9136 (52.33), 260.3137 (35.78), 241.9785 (7.15), 177.0405 (67.28), 176.0315 (100), 175.0285 (39.89, 147.0125 (59.59), 144.9953 (7.64), 135.0403 (25.52), 133.0030 (19.26), 121.0062 (71.11), 112.9499 (11.31), 107.0064 (18.36), 94.9993 (13.64), 81.9705 (14.46), 78.9489 (15.17), 77.9388 (25.15), 66.9732 (5.36), 64.9541 (2.41).

### 3.3. Determination of the in Vitro Antimicrobial Activity (Agar Disc-Diffusion Method)

Sterile filter paper discs (8 mm diameter) were moistened with the compound solution in dimethylsulphoxide of specific concentration (200 μg/disc), the antibacterial antibiotics Gentamicin and Ampicillin trihydrate (100 μg/disc) and the antifungal drug Clotrimazole (100 μg/disc) were carefully placed on the agar culture plates that had been previously inoculated separately with the microorganisms. The plates were incubated at 37 °C, and the diameter of the growth inhibition zones were measured after 24 h in the case of bacteria and 48 h in the case of *Candida albicans*.

### 3.4. Determination of the Minimal Inhibitory Concentration (MIC)

Compounds **6d**, **6e**, **6f**, **6j**, **6k** and **6l** Gentamicin and Ampicillin trihydrate were dissolved in dimethylsulphoxide at a concentration of 128 μg/mL. The twofold dilutions of the solution were prepared (128, 64, 32, …, 0.5 μg/mL). The microorganism suspensions at 106 CFU/mL (colony forming unit/mL) concentrations were inoculated to the corresponding wells. The plates were incubated at 36 °C for 24 h. The MIC values were determined as the lowest concentration that completely inhibited visible growth of the microorganism as detected by unaided eye.

### 3.5. Determination of the in Vivo Hypoglycemic Activity

Animals: Locally bred male Sprague-Dawley rats (250 ± 30 g body weight) were obtained from Abu Rawash, Giza, Egypt. The rats were housed in wire-bottomed cages at 22 ± 2 °C. A standard pellet diet and tap water were supplied *ad libitium*. The animals were acclimatized to these conditions for 15 days before the experiment.

Induction of experimental diabetes: Rats were fasted for 16 h before the induction of diabetes with STZ (Sigma Chemical Co., St Louis, MO, USA). The animals were injected intraperitoneally with 0.22–0.25 mL of a freshly prepared solution STZ (60 mg/mL in 0.01 M citrate buffer, pH 4.5) at a final dose of 60 mg/kg body weight. Only rats with serum glucose levels greater than 250 mg/dL were used in experiments.

Design of the experiment: Uniform suspensions of the compounds **6a**, **6c**, **6f**, **6g**, **6j** and **6l** and the oral hypoglycemic drug gliclazide (positive control) in 0.5% (*w*/*v*) aqueous carboxymethyl cellulose (CMC) solution were prepared at specific concentrations of 10 mg/mL in case of the test compounds and gliclazide. 48 h post STZ injection, the hypoglycemic activity of the compounds **6a**, **6c**, **6f**, **6g**, **6j** and **6l** was assessed, the diabetic rats were fasted for 16 h and divided into 14 groups each of 5 animals (*n* = 5) and the serum glucose level was determined for each group and considered as initial fasting serum glucose (C_0_). Group 1, which served as the negative diabetic control group, received only a single oral dose of 0.5% (*w*/*v*) aqueous CMC solution (5 mL/kg). Groups 2 was treated with 10 mg/kg gliclazide in 0.5% (*w*/*v*) aqueous CMC (positive control). Groups 3–14 were treated with either a single oral dose of the 10 or 20 mg/kg of the test compounds. All treatments were administered by oral gavage. 24 h after treatment, the blood samples were collected and the serum glucose level (C_24_) was determined for each group.

Determination of serum glucose: Blood samples from the tail vein were collected, allowed to clot, and centrifuged at 2000 rpm for 10 min. The serum was separated and used on the same day for the measurement of serum glucose levels using a commercial glucose oxidase (GO) assay kit (Sigma-Aldrich). Blood glucose levels were expressed in mg/dL as mean ± standard error of mean (SEM). The data were statistically analyzed using ANOVA with Tukey’s multiple comparison test. The values of *p* < 0.01 were considered as significant. The percentage of serum glucose reduction for each group was calculated in relation to the initial serum glucose level as follows:

% Serum glucose reduction = [(C_0_−C_24_/C_0_)] × 100

where C_0_ is the mean initial fasting serum glucose level, C_24_ is the mean serum glucose level 24 h after treatment.

### 3.6. Determination of the Oral Acute Toxicity of Compounds **6a**, **6f**, **6g** and **6l**

Freshly prepared suspensions of compounds **6a**, **6f**, **6g** and **6l** in concentrations of 1%, 3%, 4%, 6%, 8% and 12% in 0.5% aqueous carboxymethyl cellulose solution were prepared. Each compound was given to six groups each of six normal albino mice of both sexes by oral intubation in doses of 250, 500, 750, 1000, 1250 and 1500 mg/kg. The percentage mortality was recorded 24 h after compound administration and the oral lethal dose LD_50_ was calculated.

## 4. Conclusions

In this study, new *N*-Mannich bases of 5-(1-adamantyl)-4-substituted-1,2,4-triazoline-3-thiones **6a**–**l** were synthesized and their *in vitro* antimicrobial activity was determined. Compounds **6d**,** 6e**, **6f**,** 6j**, **6k** and **6l** displayed potent antibacterial activity. In addition, the *in vivo* oral hypoglycemic activity of compounds **6a**, **6c**, **6f**, **6g**, **6j** and **6l** was determined in streptozotocin (STZ)-induced diabetic rats. Compounds **6a**, **6f**, **6g** and **6l** produced marked hypoglycemic activity compared gliclazide. Although, the active compounds are considered to be good candidates as newer antibacterial and hypoglycemic agents, further studies including the exploration of the mechanism of their biological activity are being undertaken.
